# Unlocking Opportunities and Overcoming Challenges in Genetically Engineered Biofortification

**DOI:** 10.3390/nu17030518

**Published:** 2025-01-30

**Authors:** Abdullah Mohammad Shohael, Jojo Kelly, Srividhya Venkataraman, Kathleen Hefferon

**Affiliations:** 1 Cell Genetics and Plant Biotechnology Lab, Department of Biotechnology and Genetic Engineering, Jahangirnagar University, Dhaka 1342, Bangladesh; amshohael@juniv.edu; 2 School of Integrative Plant Sciences, Cornell University, Ithaca, NY 14850, USA; 3 Department of Cell Systems Biology, University of Toronto, Toronto, ON M5S 3B2, Canada; 4 Department of Microbiology, Cornell University, Ithaca, NY 14850, USA

**Keywords:** food fortification, health outcomes, micronutrients, nutrition status, malnutrition, food systems, molecular farming

## Abstract

Micronutrient deficiencies affect over three billion people globally; there is a particularly severe problem with iron and zinc nutrition in developing countries. While several strategies exist to combat these deficiencies, biofortification has emerged as a powerful and sustainable approach to enhance the nutritional value of staple crops. This review examines recent advances in crop biofortification and their potential to address global nutritional challenges. We present successful case studies including iron-enriched cassava, nutrient-enhanced tomatoes, and omega-3-fortified oilseed crops, demonstrating the diverse possibilities for improving nutritional outcomes. The integration of novel plant-based protein production techniques has further expanded opportunities for sustainable nutrition. However, significant challenges remain, including complex environmental interactions, regulatory considerations, and sociocultural barriers to adoption. Economic analyses suggest biofortification offers substantial return on investment, with every dollar invested generating up to seventeen dollars in benefits through reduced disease burden. As global food security challenges intensify due to climate change, biofortified crops represent a crucial tool for improving nutritional outcomes, particularly in low- and middle-income countries. We conclude by examining emerging opportunities and future directions in this rapidly evolving field.

## 1. Introduction

Malnutrition remains one of the paramount global challenges, impacting several billion people and creating both health and socioeconomic disparities. Malnutrition has wide-ranging effects that can have a significant impact on people’s health outcomes, particularly for children, who become more vulnerable to infectious diseases. Children are particularly vulnerable to impaired cognitive development due to malnutrition, which can reduce their work productivity later in life. Negative impacts on workforce effectiveness may lead to more poverty and inequality, as well as slow economic growth for countries with malnourished populaces.

An insufficient supply of regular dietary nutrients through the food system leads to undernutrition, manifesting as stunted growth, wasting, weakened immunity, and increased susceptibility to diseases, especially among the children and pregnant women of vulnerable populations [1]; additionally, a “triple burden” of malnutrition often arises, combining standard malnutrition, micronutrient deficiencies (often referred to as hidden hunger), and overnutrition, including obesity [2]. This paradox emerges when cheap, calorie-dense but nutrient-poor foods dominate diets due to economic constraints and poverty, and a lack of education and awareness leads to deficiency within the populations. Addressing malnutrition requires targeted interventions to ensure the availability and affordability of diverse, nutrient-rich foods alongside awareness initiatives to promote balanced diets [3].

Beyond these immediate challenges, the nutritional value of our food supply is declining at a concerning rate. Studies tracking the mineral content in fruits and vegetables have documented a consistent decline over time. Analysis from 1963 to 1992 revealed mineral content declines for calcium (29%), magnesium (21%), potassium (6%), phosphorus (11%), and iron (32%) [4]. More recent research through 2019 confirmed this trend, showing further reductions in sodium (52%), iron (50%), copper (49%), and magnesium (10%) [5]. This decline stems primarily from modern agriculture’s focus on developing high-yielding cultivars rather than maintaining nutritional quality.

This article describes the issues related to crop biofortification for the improvement of human health. First, key genetic engineering technologies are described, then several examples, including GABA (gamma-aminobutyric acid)-producing tomatoes, vitamin A-producing golden rice, and omega-3 oil seed crops are provided. The novel expression of animal proteins by plant molecular farming is summarized. The review concludes with a description of challenges and opportunities that genetically engineered biofortified crops can provide.

## 2. Common Crop Biofortification Schemes

Biofortification strategies encompass three main approaches: modern biotechnology (including genetic engineering, genome editing, and mutation breeding), conventional breeding, and agronomic practices such as foliar nutrient application and targeted fertilization. Through modern biotechnology, genes of interest that are involved in nutritional improvement are directly manipulated. Conventional breeding selects and crossbreeds plants with high natural levels of desired nutrients over generations to produce more nutritious varieties, such as iron-rich beans and zinc-enriched wheat [6]; however, this method is not always feasible.

**Figure 1:**
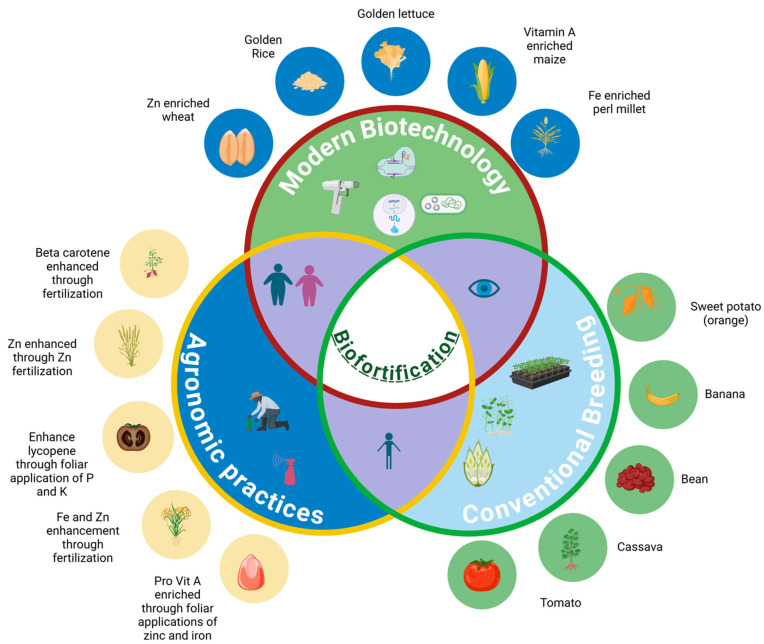
Overview of three major biofortification strategies to enhance nutritional contents in various crops (Created in BioRender.com).

Agronomic practices enhance the nutritional profile of crops through cultivation optimization. This typically includes the application of specific fertilizers or soil amendments rich in vital minerals to improve the nutrient density of edible plant parts [7]. Foliar and other applications of targeted minerals boost the levels of essential micronutrients in the edible portions of crops, making them more nutritious for human consumption [8]. This approach leverages soil and plant interactions to naturally enhance the micronutrient content (Figure 1).

## 3. Plant Genetic Engineering as a Strategy for Biofortification

Genetically engineered crops have been commercially available for the past 30 years. While the first generation of transgenic crops focused on improving farmers’ livelihoods, such as crop yield and resistance to biotic and abiotic stresses, the next generation of crops have targeted consumers themselves, by improving the variety, taste, and nutritional content of crops [9]. These crops have been developed in conjunction with improvements in traditional genome technology, so that genes directly associated with nutrient synthesis, transport, and storage can be identified. New abilities to determine how to improve the bioavailability of nutrients in crops have in turn opened the door to increased micronutrient bioavailability (such as iron and zinc or vitamin A) in a variety of staple crops [10].

While transgenic crops (expressing heterologous genes) have faced significant regulatory and public acceptance challenges, genome editing technologies have generally encountered less resistance. Many countries have approved the incorporation of single or a small number of nucleotide substitutions, additions, or deletions (SDN1 and SDN2 events) without difficulty, whereas CRISPR events that require SDN3 (addition of heterologous sequences using a genome editing approach) are still prohibited in many countries [11].

Increasingly, plant breeders are adopting genome editing technology as a quick, precise, efficient, and affordable method of modifying crops. Moreover, plant scientists can apply this knowledge to select useful genes in crops such as maize, canola and wheat for gene editing towards developing novel, enhanced varieties. Using gene editing, genes already occurring in the plant can be switched on and switched off as desired. This would be beneficial for agriculture as it would mean the rapid availability of novel crop varieties with low seed prices. A market rush has started for gene edited crops, as regions and nations brace themselves to participate in potential enhancements in crop performance and profits [12].

## 4. Examples of Biofortified and Nutritionally Enhanced Crops

### 4.1. Nutritionally Enhanced Tomatoes

Agribusiness giant Bayer has delved into the development of nutritionally enhanced crops. One new product includes a variety of vitamin D3-biofortified tomatoes, generated using genome editing. Vitamin D deficiency is common on a global scale due to limited sunlight during the winter season. In collaboration with Pairwise, Bayer has developed genome-edited mustard greens with reduced pungency while maintaining the nutrient, antioxidant, and fiber contents. These modified varieties retain their nutritional properties while potentially increasing consumer acceptance [13,14].

**Figure 2:**
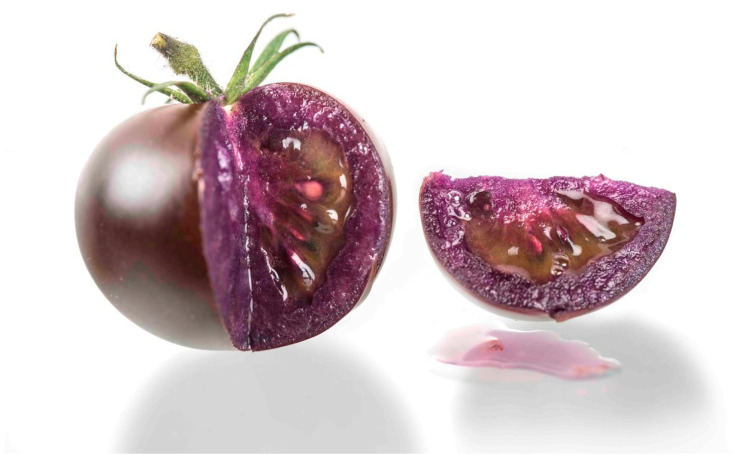
The Purple Tomato developed by Norfolk Healthy Produce [17].

The genomes of crops can also be changed so that they produce pigments, such as the anthocyanins that give carrots their orange color and blueberries their blue color. Years ago, a transgenic tomato variety that expresses high levels of anthocyanins was found to reduce the incidence of cancer in a rat model [15,16]. Today, transgenic Purple Tomatoes, produced by the company Norfolk Healthy Produce, express two snapdragon genes that give them their characteristic color (Figure 2). The purple tomatoes are packed with powerful, nutritionally beneficial anthocyanins, the same compounds found in blueberries, purple cabbages, and plums. The seeds are available for gardeners to purchase right now (Figure 2).

The Japanese company, Sanatech Seed Co., Ltd. has developed the “Sicilian Rouge High GABA” tomato using genome editing [17]. GABA (γ-aminobutyric acid) functions as a bioactive compound that has demonstrated blood-pressure-lowering effects in human studies [18]. Investigators identified the SlGAD3 gene as being critical for the accumulation of GABA in fruits [19]. They deleted the SlGAD3 autoinhibitory domain (AID), which led to the enhancement of GABA accretion in fruit [20]. They introduced a stop codon mutation preceding the nucleotide sequence that encodes the AID of the endogenous SlGAD3 using CRISPR/Cas9 technology, which led to high concentrations of GABA in these tomatoes. In actuality, the company’s commercial variety of tomato, “Sicilian Rouge”, was genetically modified to accrue high GABA levels. The company now delivers these GABA-enriched tomatoes to consumers directly. This launch of the first genome-edited tomatoes in Japan is expected to have a major impact on the advancement trend of genetically engineered crops. Japan was the first nation in the world to introduce an unprocessed genome-edited crop into the market and presently leads the world in the social application of this technology. The fruitful launch of high-GABA tomatoes has had a significant impact on advancing the rules of handling such GE crops on a global scale.

### 4.2. β-Carotene Biofortification

Vitamin A deficiency (VAD) remains a critical global health challenge and particularly affects children and pregnant women in developing regions, potentially causing blindness and weakened immune systems [1]. To address this issue, researchers have developed several biofortification strategies targeting different crops to enhance their β-carotene content, a precursor to vitamin A.

Golden Rice represents one of the earliest and most well-known successes in β-carotene biofortification. This genetically engineered variety produces beta-carotene within the rice grain through the synergistic action of two inserted genes, phytoene synthase (*psy*) from maize (*Zea mays*) and carotene desaturase (*crtI*), from the soil bacterium *Erwinia uredovora* [21]. Studies have validated that Golden Rice serves as an effective and bioavailable source of pro-vitamin A [22,23].

Recent advances have expanded β-carotene biofortification to other crops. At the Spanish National Research Council (CSIC), researchers led by Manuel Rodríguez-Concepción developed an innovative approach for green leafy vegetables [24]. Their dual-strategy method involves producing β-carotene in leaf cell cytosol while converting chloroplasts to carotenoid-accumulating chromoplasts through the introduction of bacterial phytoene synthase (*crtB*). When combined with optimized light conditions, this technique achieved remarkable results—a 30-fold increase in both β-carotene content and bioavailability [25]. This breakthrough demonstrates how combining genetic engineering with environmental optimization can enhance nutritional value while preserving crop performance.

CRISPR/Cas9 technology has further expanded biofortification possibilities, as demonstrated in banana improvement [26]. Researchers successfully enhanced β-carotene levels in the Grand Naine Cavendish cultivar by editing the lycopene epsilon-cyclase gene. This modification resulted in a significant 6-fold increase in β-carotene content within the fruit pulp compared to unedited plants [26], illustrating the potential of precise genome editing in nutritional enhancement.

### 4.3. Omega 3 Biofortifiaction

It is well known that omega-3 polyunsaturated fatty acids play important roles in several aspects of human health, with fish currently serving as the primary dietary source. However, declining global fish stocks have motivated the development of sustainable, plant-based alternatives. Through genetic engineering, researchers have successfully “reverse-engineered” crops to produce these beneficial fatty acids, creating products suitable for human consumption, fortified foods, infant formula, and animal feed [27].

The seed oil crop *Camelina* (false flax) has been engineered to produce fish fatty acids, such as omega-3 fatty acids EPA and DHA. These omega-3 LC-PUFA levels are equivalent to those in fish oils and represent a sustainable, terrestrial source of these fatty acids [28]. Building on this work, Nuseed has developed FDA-approved canola varieties that express microalgal genes mimicking natural synthesis pathways. These plants achieve a favorable omega-6 to omega-3 ratio of 1:4, with one to two hectares of cropland producing DHA equivalent to 10,000 kg of wild-caught fish [29]. Such production efficiencies promise to make essential fatty acids more accessible and affordable worldwide.

Additionally, biotech companies have developed alternative approaches to improving oil quality. Calyxt has used TALEN technology to generate high-oleic-acid soybean varieties [30], further demonstrating the potential of precision breeding for enhancing nutritional traits in oilseed crops.

### 4.4. Potato

Processed foods can be engineered to produce more healthy cooking outcomes. For example, The Innate Potato™, produced by the company Simplot, has an 8-fold reduction in acrylamide content [31]. Studies have suggested that acrylamide is carcinogenic in rodents. Innate potatoes could reduce the average daily intake of acrylamide by one-fourth [32]. Furthermore, lowering the risk of enzymatic browning in potatoes by using genome editing to create a frameshift mutation in the PPO gene is another way to help reduce the accumulation of food waste [33]. 

### 4.5. Iron-Enhanced Crops

Biotechnological approaches have been used to produce several crops with elevated levels of iron and zinc. Iron (Fe) deficiency is a global health problem, especially in underdeveloped countries. Biofortification via genetic engineering methodology has been used to improve Fe nutrition in several crops. Various steps, e.g., uptake, distribution, and storage, involved in Fe homeostasis have been manipulated to increase the Fe concentration in the edible portions of plants. The following is a list of assorted food crops that have been genetically engineered to improve iron bioavailability.

### 4.6. Bean

Beans are a significant food source across the globe. The genetic engineering of beans to improve iron and zinc accumulation from uptake in the soil to storage in the seed is under examination. Increasing the bioavailability of iron by increasing ferritin levels and decreasing levels of the antinutrient phytate is currently being investigated [34].

### 4.7. Cassava

Cassava is consumed by over 800 million people across the globe; more specifically, sub-Saharan populaces require cassava for over half of their calories. Cassava, however, is low in micronutrients such as iron and zinc, and the scarcity of genetic variation within the germplasm does not enable facile improvement of these micronutrient densities using traditional plant breeding techniques. As a result, transgenic cassava plants have been developed to contain increased iron and zinc concentrations by expressing a mutant version of the iron transporter protein (IRT1) and ferritin (FER1) from *Arabidopsis*. These transgenic cassava plants were able to accumulate iron levels 7–18 times higher and zinc levels 3–10 times higher, adequately reaching the levels needed in the human diet [35].

### 4.8. Rice

Rice, a major staple food crop, currently feeds more than half the world’s population. The endosperm of white rice is particularly low in iron and zinc, and thus people in low- and middle-income countries are prone to deficiencies of these micronutrients. As a result, much research has focused on the use of transgenic biofortification techniques to improve iron and zinc bioavailability in rice. Alterations in the expression of ferritin genes and metal transporters and changes in the nicotianamine/phytosiderophore pathway (including biosynthetic genes and transporters), regulators of iron deficiency responses, and others were found to increase iron and zinc in rice seed. Further examination of iron and zinc yields under field conditions, as well as genes manipulated by genome editing, are also necessary [36].

Another way to improve rice’s micronutrient status could be to incorporate better performing genes within the rice plants. For example, the expression of ferritins from kidney bean and pearl millet alongside rice nicotianamine synthase 2 (*OsNAS2*) in transgenic rice endosperm improved iron content [37]. More specifically, these transgenic lines demonstrated the potential to surpass 30% of the estimated average requirement (13 μg/g Fe and 28 μg/g Zn) proposed for rice in the HarvestPlus breeding program.

Iron-biofortified rice was developed through two complementary strategies: (1) expression of YELLOW STRIPE 1 under the HEAVY METAL ATPASE 2 promoter, which increased iron concentrations 4.8-fold compared to non-transgenic plants, and (2) expression of the mugineic acid transporter under the ferric reductase defective like 1 promoter control, yielding 3.2-fold higher iron levels. While combining these transgenes did not produce synergistic effects on iron concentration, it enhanced polished grain iron content by 5.1- to 9.3-fold [38].

### 4.9. Wheat

Wheat biofortification efforts have demonstrated success in both laboratory and field conditions. Multi-year field trials of bread wheat expressing rice nicotianamine synthase 2 (*OsNAS2*) showed sustained improvements in iron and zinc bioavailability across both whole and white flour products. These improvements resulted from enhanced biosynthesis of the iron transport compounds nicotianamine (NA) and 2′-deoxymugineic acid (DMA) [39,40].

**Figure 3:**
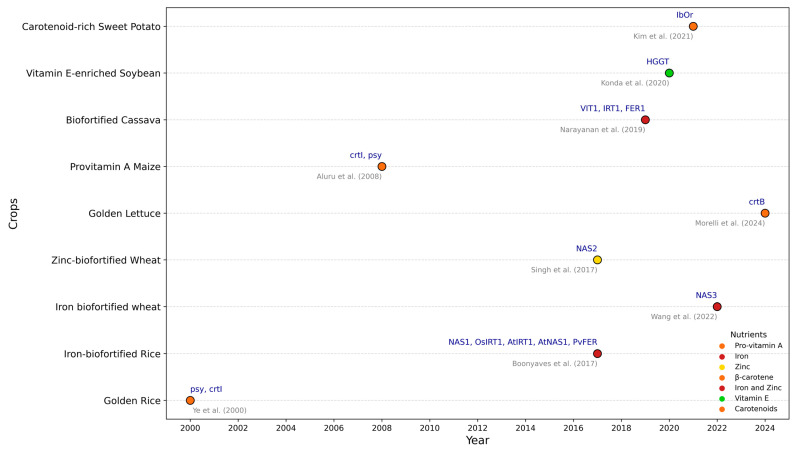
Overview of the bioengineered crops highlighting the intersection of genetic innovation and nutritional outcomes [25,35,41,42,43,44,45,46].

Complementary approaches using wheat’s own genetic resources have also proven effective. Expression of *Triticum monococcum* nicotianamine synthase 3 (*TmNAS3*) doubled iron accumulation in wheat, with studies demonstrating that careful regulation of synthase expression can optimize iron enhancement [41]. These findings are particularly significant, as they demonstrate the potential for iron biofortification in a crop that provides 20% of global caloric intake (Figure 3).

## 5. Molecular Farming for Animal Proteins Using Plant Platforms

As a different approach to improving nutritional status, molecular farming has been utilized as a process to generate nutritionally valuable animal proteins in plants. Molecular farming has been used for decades to produce cheap pharmaceuticals for developing countries [47]. At this point, plant biotechnology has become advanced enough to also consider the production of animal food products in plants without using any animals.

Global demand for animal products is projected to increase by 70% between 2014 and 2050 [48]. Molecular farming could help meet this growing demand while offering several advantages. This technology creates animal products without raising livestock, which improves animal welfare and reduces both land use and greenhouse gas emissions. The resulting products are free from the hormones and antibiotics commonly used in traditional animal agriculture. Additionally, molecular farming could provide nutritious alternatives for people who lack access to animal products or avoid them for religious reasons. From a farmer perspective, the inclusion of animal products in the form of molecular farming can add more value to products they are growing in the field or greenhouse [49].

For example, the companies Moolec and Nobell Foods (now known as Alpine Bio) are generating transgenic soybeans that expresses myoglobin and casein, respectively. Farmers who grow these crops will be able to tap into an additional market stream to produce alternative proteins for the food industry. Soybeans are used in many food products. Myoglobin, when produced in soy, could be used to improve the iron status of consumers while helping them to avoid animal products. Casein could be used for cheese products without the use of animals as well [50].

Other molecular farming companies are also on the rise in the field of alternative food proteins for improved diets. In Israel, a company known as Palopo makes chicken egg albumin in transgenic potato plants, and the company BioBetter produces animal growth factors for cultivated meat in transgenic tobacco plants. The advancements made in this field can have a positive effect for low- and middle-income countries by enabling inexpensive, sustainable, plant-based technologies to address malnutrition and food security challenges for disadvantaged populaces.

## 6. Biochemical Studies on Nutrient Biofortification in Crops

CRISPR-Cas9 editing was used to knock out the gene Inducer Silencing of Anthocyanins in Cell Cultures (StlSAC) in cell cultures of the “Blue Star” variety of potato. This led to deletions in this gene that resulted in a frame-shift mutation and consequent loss of function, leading to the development of dark purple pigmentation against the mosaic white color in the potato wild type. The edited potato cells were shown to have 3-fold increased anthocyanin content when compared to the wild type and about 14-fold higher anthocyanin content than that of potato tubers [51].

In Tartary buckwheat, a polycistronic tRNA-SgRNA-enabled CRISPR-Cas9 editing was used to knock out the gene FtMYB45, which resulted in enhanced contents of eight flavonoids, including anthocyanin and proanthocyanidin [52]. Through CRISPR-based mutagenesis, it was shown in Malus domestica that a knock-out mutation in the MdMYB16 gene led to a doubling of the anthocyanin content [53]. The pYLCRISPR/Cas9 multiplex construct containing three SgRNAs was used to knock out the gene PtrMYB57, which codes for the R2R3-MYB anthocyanin repressor in poplar. These mutants produced a 2-fold higher anthocyanin content when compared to that of the wild type [54].

CRISPR-Cas9 mutagenesis was used to knock out the SlMYBATV gene in tomatoes, which resulted in a 12-fold higher anthocyanin content than that in the wild type [55]. Further, the fruit-specific promoter-based expression of the functioning SlAN2-like gene in a tomato cultivar resulted in the enhancement of the expression levels of the entire anthocyanin biosynthesis pathway and several-fold accretion of anthocyanins in tomato flesh and peel.

In the rice endosperm, anthocyanin biosynthesis was achieved using a highly efficient multi-transgene stacking vector system. Eight genes related to anthocyanin biosynthesis, inclusive of two regulatory genes from maize, namely, ZmPl (R2R3-MYB TF) and ZmLc (bHLH TF), and six structural genes SsANS, SsDFR, SsF3H, SsF3H, SsCHI, and SsCHS from Coleus were introduced into the rice plants under the control of the rice endosperm-specific promoters. The resulting plants, called “Purple endosperm Rice”, contained the highest anthocyanin content by 100 mg per 100 g of the dried grain, in addition to improvement in antioxidant activities when compared to the colorless rice lines [56]. Purple maize rich in anthocyanin in the endosperm and embryo was generated by overexpressing the genes ZmR2, ZmC1, ZmBz2, and ZmBz1 using modified promoters that were tissue specific. This transgenic maize contained total anthocyanin levels as high as 291 mg/100 g of maize [57]. In another study, knock out of a VvbZIP36 allele in grapevine resulted in the accretion of anthocyanins and flavonoids such as naringenin, cyanidin-3-O-glucoside, dihydroflavonols, and chalcone [58].

The SlGABA-T1 gene was targeted in tomato using an RNA interference strategy under the regulation of the constitutive CaMV 35S promoter in the tomato Micro-Tom. These suppressed 35S::SlGABA-T1RNAi lines contained 1.3-fold higher GABA levels (118.6 mg/100 g FW) compared to the wild type at the green mature stage, 2-fold greater GABA content at the yellow stage (126.8 mg/100 g FW), and 6.8-fold more at the matured stage (106.2 mg/100 g FW) [59]. Suppression of SlGABA-T1 decreased the GABA catabolism to succinic semialdehyde (SSA) at the ripening stage, minimizing its degradation from the heights reached at the breaker level. In a different approach, a multiplexed CRISPR-Cas9 vector containing six gRNA cassettes was used to target the three SlGABA-Ts in tomato, along with CAT9 and SlSSADH, which were transformed in the tomato Ailsa Craig cultivar. Among these, the SlGABA-T1-edited tomato lines exhibited 1.43-fold greater GABA levels (102.80 mg/100 g FW) compared to the wild type at the green mature stage and 2.95-fold higher GABA levels at the red stage (73.83 mg/100 g FW) [60].

In tomato, sgr1 null lines generated by CRISPR-Cas9 editing accrued higher levels of the flavor-eliciting total carotenoids and ascorbic acid than the wild type. Additionally, the GABA content and total flavonoid, 2,2-diphenyl-1-picrylhydrazyl (DPPH) radical, and total phenolic levels of the sgr1 null lines were elevated compared to the wild type. Hence, the CRISPR-Cas9-based knock out of the SGR1 gene improved several functional substances in tomato fruit, thus fulfilling the antioxidant characteristics essential to consumers [61]. Rice fortified with GABA was generated using CRISPR-Cas9 editing in a pioneering effort that resulted in the truncation of the C-terminus of the OsGAD3 coding region; this modified rice showed 7-fold greater GABA content in addition to notably higher protein content and grain weight compared to the brown wild-type rice [62]. “Sicilian Rouge High GABA” tomatoes were generated by gene editing; these plants were shown to accrue 4–5 times more GABA compared to ordinary tomatoes. This involved targeted deletion of the glutamate decarboxylase C-terminus [63].

The biosynthesis of ascorbic acid and metabolism of carotenoids are controlled by EIL2 (ETHYLENE-INSENSITIVE 3-LIKE) in tomato [64]. Compared to red fruits in the wild-type tomato 45 days following pollination, the fruits of SlEIL2 RNA interference lines and the CRISPR/Cas9 eil2 mutants showed orange or yellow fruits that promoted their nutritional quality and commercial value. The CRISPR-Cas9 system was used to increase the beta-carotene content in the banana “Grand Naine” cultivar through editing of the lycopene epsilon-cyclase gene, which resulted in a significant 6-fold increase in the accumulation of beta-carotene (~24 μg/g) in the fruit pulp when compared to the non-edited banana plants [65].

In soybean, the Fatty Acid Desaturase 2 (GmFAD2) gene, which is involved in the conversion of monounsaturated oleic acid (C18:1) to polyunsaturated linoleic acid (C18:2), was mutated using CRISPR-Cas9 [66]. Analysis of the fatty acid profiles of the T1 seeds obtained from the CRISPR-edited soybean that is homozygous for both the GmFAD2 genes demonstrated notable augmentation of the oleic acid levels to over 80%, whereas the linoleic acid content reduced to 1.3–1.7%. Additionally, transgene-free soybeans high in oleic acid content were generated as quickly as the T1 generation.

In pennycress (*Thlaspi arvense* L.), CRISPR-Cas9 technology was used to generate knock out mutations in the REDUCED OLEATE DESATURATION1 (ROD1) and FATTY ACID DESATURASE2 (FAD2) genes, which led to enhanced oleic acid content [67]. Oil containing high oleic acid (18:1) is highly desirable due to its oxidative stability, which is superior to that of polyunsaturated fatty acids (PUFAs) linoleic acid (18:2) and linolenic (18:3), in addition to its augmented cold-flow characteristics. The above rod1 fae1 and fad2 fae1 double mutants were combined with the knockout of the FATTY ACID ELONGATION1 (fae1) gene, which showed 60% and 90% oleic acid in the seed oil, respectively. The PUFA levels also decreased to below 5%.

The OsNAS2 promoter in rice was edited using CRISPR-Cas9 through the deletion of the ARR1AT cis-regulatory element at the position −933, which led to increased Zn accumulation in the grain and in the plant. This also augmented the spikelet number for each main panicle, which resulted in more grain per plant. Further, these inherited traits were homozygous and transgene-free [68]. Targeted editing of the genes arsenite tolerant 1 (*astol1*) and Vacuolar Tron Transporter (*VIT*) in rice led to higher selenium and iron levels, respectively, affording better nutritional value [69]. Phytic acid is a major antinutrient occurring in cereal grains that decreases the bioavailability of zinc and iron in the human body, leading to malnutrition. CRISPR-Cas9 was used to mutate the *Inositol pentakis phosphate2 kinase1* (*TaIPK1*) gene in wheat. The TaIPK1.A expression was abundant in the early grain filling stages and upon gene editing; this proved to lower the phytic acid content and augment the zinc and iron accumulation in the grains compared to the wild-type plants [70].

## 7. Opportunities and Challenges of Genetically Engineered Biofortified Crops

### 7.1. Opportunities

Biofortified crops offer numerous opportunities to improve global health. Most immediately, they allow staple foods to be enhanced with essential nutrients like iron and zinc, directly addressing common deficiencies. This approach is particularly promising as it integrates seamlessly into existing diets and food systems—farmers simply plant improved varieties, eliminating the need for significant behavioral changes from consumers.

Economically, biofortification represents a worthwhile investment for addressing micronutrient deficiencies through existing agricultural systems. According to the Copenhagen Consensus, investing in micronutrient deficiency reduction through biofortification delivers impressive economic returns—for each USD 1 invested, USD 17 is generated in decreasing nutrition-related disease burden [71]. Consistently, cost-effectiveness analyses show biofortification outperforming alternative interventions like supplementation and fortification in most scenarios [72]. The Rwanda iron bean program illustrates biofortification’s economic potential through RWR2245, which delivered 20–49% higher yields than traditional varieties while providing double the iron content (82.5 mg/kg vs. 47.5 mg/kg). The variety enhanced household food security, increased market participation with 12% higher likelihood of sales, and achieved widespread adoption, reaching 28% of rural households between 2012 and 2015 [73].

Molecular farming presents opportunities to reduce the cost of producing animal proteins, with a 2019 technoeconomic analysis estimating production costs at USD 3.00–6.88/g, though recent industry data suggest significant improvements [74]. Beyond enhancing food security and economic opportunities, this technology complements cultivated meat production, where animal cells are grown in controlled environments without slaughter. While cultivated meat could have a 92% reduced impact on global warming compared to conventional beef, its adoption is limited by the high cost of the growth factors and proteins needed for cellular agriculture [75]. Molecular farming could lower these costs, making cultivated meat more accessible while providing consumers with antibiotic-free, hormone-free protein options that can be nutritionally optimized for reduced cholesterol and saturated fat content.

### 7.2. Challenges

To tackle global malnutrition challenges, biofortified crops enriched with necessary micronutrients (Fe, zinc, vitamin A) offer a sustainable solution to the traditional fortification program. New breeding techniques are promising alternatives to achieving rapid and target amounts of desirable nutrients in staples. Progress towards achieving the goals is now hindered due to public acceptance, regulatory hurdles, and policy restrictions [76].

Regulatory frameworks present significant barriers to biofortified crop deployment in regions with urgent nutritional needs. There is a worldwide “regulatory slowdown” in GM crop approvals, despite their potential to address deficiencies affecting two billion people [77]. The case of Golden Rice illustrates these challenges, i.e., waiting 15 years for regulatory approval despite widespread vitamin A deficiency. This regulatory burden is particularly severe in Africa, where frameworks are costly, lack transparency, and are extremely risk-averse, creating a paradox where regulatory caution potentially outweighs the documented impacts of existing nutritional deficiencies, with only eleven of fifty-four nations approving GM crop cultivation [78].

Historical and socio-cultural dynamics have significantly influenced biofortified crop adoption in low- and middle-income countries (LMICs), where they interact with practical factors like seed availability and yield [79]. In southern Africa, yellow maize adoption has faced resistance due to historical associations with food aid and perceptions of being “poor people’s food” [80]. One study in Kenya found only 26% of consumers would buy yellow maize at the same price as white maize [81]. These cultural barriers can be overcome through targeted interventions—for instance, in Rwanda, iron-biofortified beans achieved 29% adoption through programs that combined agricultural performance messaging with nutrition education [82]. Studies demonstrate that consumer acceptance increases significantly when communities are informed about nutritional benefits and when varieties are developed in accordance with local preferences and traditional dietary patterns [83]. Successful adoption thus requires addressing both socio-cultural barriers and practical constraints through well-designed delivery programs that engage local communities and respect traditional food preferences.

Several significant technical challenges complicate crop biofortification implementation. A key barrier is genotype × environment (G × E) interaction—for example, in common beans, locational and seasonal factors explain nearly as much phenotypic variation in zinc concentration (26.2%) as genotype (28.0%) [84]. Soil nutrient deficiency presents another critical challenge—in India alone, 36.5% of agricultural land is zinc deficient [85]. Post-harvest stability poses an additional hurdle, particularly for vitamins. Studies in Zambia showed that β-carotene levels in biofortified corn dropped by 70% after 6 months of storage [86]. These technical barriers are further complicated by the need to simultaneously maintain desirable agronomic traits while achieving targeted nutrient levels.

## 8. Fate of Biologically Active Compounds in Biofortified Crops

Bioactive compounds are crucial for human health because they reduce oxidative stress, modulate inflammation, and prevent chronic diseases [87]. The biofortification of crops, whether through conventional breeding, agronomic practices, or genetic engineering, can influence these compounds positively or negatively. While biofortification aims to increase specific micronutrients, such as iron, zinc, or provitamin A, the metabolic changes induced can impact the synthesis and accumulation of other biologically active compounds. For instance, introducing genes for carotenoid biosynthesis may lead to metabolic trade-offs that reduce polyphenol levels or alter antioxidant activity [88]. Similarly, environmental factors like light, soil composition, and post-harvest storage conditions further affect nutrient stability and bioavailability [89].

## 9. A Regulatory Milestone in Promoting Biofortification Research

Site-directed nuclease (SDN) genome editing approaches, particularly SDN1 and SDN2 methods, which involve precise genetic modifications without introducing foreign DNA, have been deregulated in countries like Bangladesh, the United States, and Japan, as they align with natural genetic variations [90]. This deregulation process encourages innovation and the development of nutrient-rich crops through this technology.

Public hesitation toward genetically engineered (GE) crops often stems from insufficient awareness, mistrust, long regulatory processes, and limited communication about the benefits and risks [91]. Effective stewardship planning, enhancing communication using scientific evidence, and improving science education can address misconceptions and the fear of unethical interventions, thereby improving community perception. For sustainable biofortification, though the genetic engineering sector will leverage nutritional improvement, it is essential to educate the public, enabling them to make informed decisions [92].

## 10. Conclusions

The production of biofortified crops continues to maintain tremendous appeal and support from the scientific community. As global populations grow in an increasingly warmer world, the production of novel varieties of biofortified crops with enhanced nutritional benefits should be predicted to receive a warm welcome, particularly in LIMCs. While the genetic engineering of transgenic plants continues to follow an onerous path toward regulatory approval and consumer acceptance, genome editing as a technology appears to be advancing without similar hindrances. It is our hope that future research and development concerning nutritionally enhanced crops will improve the health and well-being of many, as well as lower the potential for environmental damage. This could be achieved within the next two decades through the improvement of crop yield and nutritional content, as well as using technologies such as molecular farming to replace animal-sourced protein and thus reduce the use of animals in agriculture.

## References

[1] UNICEF (2020). The State of the World’s Children 2020: Nutrition, for Every Child.

[2] Lowe N., Lowe N., Qualter P., Sinclair J., Gupta S., Zaman M. (2023). School Feeding to Improve Cognitive Performance in Disadvantaged Children: A 3-Arm Parallel Controlled Trial in Northwest Pakistan. Nutrients.

[3] Food and Agriculture Organization (FAO) (2019). The State of Food Security and Nutrition in the World 2019.

[4] Jack A. (1998). Nutrition Under Siege. One Peaceful World.

[5] Bhardwaj R.L., Parashar A., Parewa H.P., Vyas L. (2024). An alarming decline in the nutritional quality of foods: The biggest challenge for future generations’ health. Foods.

[6] Pfeiffer W.H., McClafferty B. (2007). Biofortification: Breeding micronutrient-dense crops. Breeding Major Food Staples.

[7] Datta K., Datta S.K., Wang K. (2006). Indica rice (Oryza sativa, BR29 and IR-64). Methods in Molecular Biology: Agrobacterium Protocols.

[8] Ierna A., Pellegrino A., Mauro R.P., Leonardi C. (2020). Micronutrient Foliar Fertilization for the Biofortification of Raw and Minimally Processed Early Potatoes. Agronomy.

[9] Yang Y., Xu C., Shen Z., Yan C. (2022). Crop Quality Improvement Through Genome Editing Strategy. Front. Genome Ed..

[10] Kumar D., Yadav A., Ahmad R., Dwivedi U.N., Yadav K. (2022). CRISPR-Based Genome Editing for Nutrient Enrichment in Crops: A Promising Approach Toward Global Food Security. Front. Genet..

[11] Buchholzer M., Frommer W.B. (2022). An increasing number of countries regulate genome editing in crops. New Phytol..

[12] Financial Times (2024). Proposed EU Ban On Gene-Edited Crop Patents Prompts Dispute. https://www.ft.com/content/5e3cc871-1377-4c65-88e5-e9096fb4db1f.

[13] Li J., Scarano A., Gonzalez N.M., D’orso F., Yue Y., Nemeth K., Saalbach G., Hill L., Martins C.d.O., Moran R. (2022). Biofortified tomatoes provide a new route to vitamin D sufficiency. Nat. Plants.

[14] Karlson D., Mojica J.P., Poorten T.J., Lawit S.J., Jali S., Chauhan R.D., Pham G.M., Marri P., Guffy S.L., Fear J.M. (2022). Targeted Mutagenesis of the Multicopy Myrosinase Gene Family in Allotetraploid Brassica juncea Reduces Pun-gency in Fresh Leaves across Environments. Plants.

[15] Butelli E., Titta L., Giorgio M., Mock H.P., Matros A., Peterek S., Schijlen E.G.M., Hall R.D., Bovy A.G., Luo J. (2008). Enrichment of tomato fruit with health-promoting anthocyanins by expression of select transcription factors. Nat. Biotechnol..

[16] Gonzali S., Perata P. (2020). Anthocyanins from Purple Tomatoes as Novel Antioxidants to Promote Human Health. Antioxidants.

[17] Ezura K., Nakamura A., Mitsuda N. (2022). Genome-wide characterization of the TALE homeodomain family and the KNOX-BLH interaction network in tomato. Plant Mol. Biol..

[18] Yoshimura S., Gerondopoulos A., Linford A., Rigden D.J., Barr F.A. (2010). Family-wide characterization of the DENN domain Rab GDP-GTP exchange factors. J. Cell Biol..

[19] Takayama H., Landes E., Truby L., Fujita K., Kirtane A.J., Mongero L., Yuzefpolskaya M., Colombo P.C., Jorde U.P., Kurlansky P.A. (2015). Feasibility of smaller arterial cannulas in venoarterial extracorporeal membrane oxygenation. J. Thorac. Cardiovasc. Surg..

[20] Takayama M., Matsukura C., Ariizumi T., Ezura H. (2017). Activating glutamate decarboxylase activity by removing the autoin-hib-itory domain leads to hyper γ-aminobutyric acid (GABA) accumulation in tomato fruit. Plant Cell Rep..

[21] Al-Babili S., Hoa T.T., Schaub P. (2006). Exploring the potential of the bacterial carotene desaturase CrtI to increase the beta-carotene content in Golden Rice. J. Exp. Bot..

[22] Ye X., Al-Babili S., Kloti A., Zhang J., Lucca P., Beyer P., Potrykus I. (2000). Engineering the provitamin A (β-carotene) biosynthetic pathway into rice endosperm. Science.

[23] Tang G., Qin J., Dolnikowski G.G., Russell R.M., Grusak M.A. (2009). Golden Rice is an effective source of vitamin A. Am. J. Clin. Nutr..

[24] Paine J.A., Shipton C.A., Chaggar S., Howells R.M., Kennedy M.J., Vernon G., Wright S.Y., Hinchliffe E., Adams J.L., Silverstone A.L. (2005). Improving the nutritional value of Golden Rice through increased pro-vitamin A content. Nat. Biotechnol..

[25] Morelli L., Perez-Colao P., Reig-Lopez D., Di X., Llorente B., Rodriguez-Concepcion M. (2024). Boosting pro-vitamin A content and bi-oaccessibility in leaves by combining engineered biosynthesis and storage pathways with high-light treatments. Plant J..

[26] Kaur N., Alok A., Shivani Kumar P., Kaur N., Awasthi P., Chaturvedi S., Pandey P., Pandey A., Pandey A.K., Tiwari S. (2020). CRISPR/Cas9 directed editing of lycopene epsilon-cyclase modulates metabolic flux for β-carotene biosynthesis in banana fruit. Metab. Eng..

[27] Venegas-Calerón M., Sayanova O., Napier J.A. (2010). An alternative to fish oils: Metabolic engineering of oil-seed crops to produce omega-3 long chain polyunsaturated fatty acids. Prog. Lipid Res..

[28] Ruiz-Lopez N., Haslam R.P., Napier J.A., Sayanova O. (2014). Successful high-level accumulation of fish oil omega-3 long-chain poly-unsaturated fatty acids in a transgenic oilseed crop. Plant J..

[29] Belide S., Shrestha P., Kennedy Y., Leonforte A., Devine M.D., Petrie J.R., Singh S.P., Zhou X.R. (2022). Engineering docosapentaenoic acid (DPA) and docosahexaenoic acid (DHA) in Brassica juncea. Plant Biotechnol. J..

[30] University of Minnesota (n.d.) The importance of Technology Transfer|Better World. https://autm.net/about-tech-transfer/better-world-project/bwp-stories/talen.

[31] Ye J., Shakya R., Shrestha P., Rommens C.M. (2010). Tuber-specific silencing of the acid invertase gene substantially lowers the acry-la-mide-forming potential of potato. J. Agric. Food Chem..

[32] Del Mar Martínez-Prada M., Curtin S.J., Gutiérrez-González J.J. (2021). Potato improvement through genetic engineering. GM Crops Food..

[33] Chincinska I.A., Miklaszewska M., Sołtys-Kalina D. (2022). Recent advances and challenges in potato improvement using CRISPR/Cas genome editing. Planta..

[34] Huertas R., Karpinska B., Ngala S., Mkandawire B., Maling’A J., Wajenkeche E., Kimani P.M., Boesch C., Stewart D., Hancock R.D. (2022). Biofortification of common bean (*Phaseolus vulgaris* L.) with iron and zinc: Achievements and challenges. Food Energy Secur..

[35] Narayanan N., Beyene G., Chauhan R.D., Gaitán-Solís E., Gehan J., Butts P., Siritunga D., Okwuonu I., Woll A., Jiménez-Aguilar D.M. (2019). Biofortification of field-grown cassava by engineering expression of an iron transporter and ferritin. Nat. Biotechnol..

[36] Wairich A., Ricachenevsky F.K., Lee S. (2022). A tale of two metals: Biofortification of rice grains with iron and zinc. Front. Plant Sci..

[37] Gupta B.B., Mishra S.K., Banoth S.K., Baliyan S., Chauhan H. (2023). Iron and zinc biofortification of rice by synergistic expression of OsNAS2 gene with monocot (*Pennisetum glaucum*) and dicot (*Phaseolus vulgaris*) ferritins. Plant Physiol. Biochem..

[38] Kawakami Y., Gruissem W., Bhullar N.K. (2022). Novel rice iron biofortification approaches using expression of ZmYS1 and OsTOM1 controlled by tissue-specific promoters. J. Exp. Bot..

[39] Zha M., Li X., Li R., Huang J., Fan J., Zhang J., Wang Y., Zhang C. (2022). Overexpression of Nicotianamine Synthase (AtNAS1) Increases Iron Accumulation in the Tuber of Potato. Plants.

[40] Beasley J.T., Bonneau J.P., Moreno-Moyano L.T., Callahan D.L., Howell K.S., Tako E., Taylor J., Glahn R.P., Appels R., Johnson A.A.T. (2022). Multi-year field evaluation of nicotianamine biofortified bread wheat. Plant J..

[41] Wang H., Liao S., Li M., Wei J., Zhu B., Gu L., Li L., Du X. (2022). TmNAS3 from Triticum monococum directly regulated by TmbHLH47 increases Fe content of wheat grain. Gene.

[42] Aluru M., Xu Y., Guo R., Wang Z., Li S., White W., Wang K., Rodermel S. (2008). Generation of Transgenic Maize with Enhanced Provitamin A Content. J. Exp. Bot..

[43] Boonyaves K., Wu T.Y., Gruissem W., Bhullar N.K. (2017). Enhanced Grain Iron Levels in Rice Expressing an Iron-Regulated Metal Transporter, Nicotianamine Synthase, and Ferritin Gene Cassette. Front. Plant Sci..

[44] Singh S.P., Keller B., Gruissem W., Bhullar N.K. (2017). Rice Nicotianamine Synthase 2 Expression Improves Dietary Iron and Zinc Levels in Wheat. Theor. Appl. Genet..

[45] Konda A.R., Nazarenus T.J., Nguyen H., Yang J., Gelli M., Swenson S., Cahoon E.B. (2020). Metabolic Engineering of Soybean Seeds for Enhanced Vitamin E Tocochromanol Content and Effects on Oil Antioxidant Properties in Polyunsaturated Fatty Acid-Rich Germplasm. Metab. Eng..

[46] Kim S.E., Lee C.J., Park S.U., Lim Y.H., Park W.S., Kim H.J., Ahn M.J., Kwak S.S., Kim H.S. (2021). Overexpression of the Golden SNP-Carrying Orange Gene Enhances Carotenoid Accumulation and Heat Stress Tolerance in Sweetpotato Plants. Antioxidants.

[47] Good Food Institute (GFI) (n.d.) Plant Molecular Farming: Key Facts. https://gfi.org/resource/plant-molecular-farming-facts/.

[48] Ivanovich C.C., Sun T., Gordon D.R., Ocko I.B. (2023). Future warming from global food consumption. Nat. Clim. Change.

[49] Schillberg S., Finnern R. (2021). Plant molecular farming for the production of valuable proteins—Critical evaluation of achievements and future challenges. J. Plant Physiol..

[50] Watson E. (2022). Proudly Genetically Modified: Moolec Molecular Farming co. Gears up to Launch Meat Proteins from GM Crops. Food Navigator-USA. https://www.foodnavigator-usa.com/Article/2022/04/25/Proudly-genetically-modified-Moolec-molecular-farming-co-gears-up-to-launch-meat-proteins-from-GM-crops.

[51] D’Amelia V., Staiti A., D’Orso F., Maisto M., Piccolo V., Aversano R., Carputo D. (2022). Targeted mutagenesis of StISAC stabilizes the production of anthocyanins in potato cell culture. Plant Direct.

[52] Wen D., Wu L., Wang M., Yang W., Wang X., Ma W., Sun W., Chen S., Xiang L., Shi Y. (2022). CRISPR/Cas9-Mediated Targeted Mutagenesis of FtMYB45 Promotes Flavonoid Biosynthesis in Tartary Buckwheat (*Fagopyrum tataricum*). Front. Plant Sci..

[53] Xu H., Wang N., Liu J., Qu C., Wang Y., Jiang S., Lu N., Wang D., Zhang Z., Chen X. (2017). The molecular mechanism underlying anthocyanin metabolism in apple using the *MdMYB16* and *MdbHLH33* genes. Plant Mol. Biol..

[54] Wan S., Li C., Ma X., Luo K. (2017). PtrMYB57 contributes to the negative regulation of anthocyanin and proanthocyanidin biosynthesis in poplar. Plant Cell Rep..

[55] Sun C., Deng L., Du M., Zhao J., Chen Q., Huang T., Jiang H., Li C.-B., Li C. (2020). A transcriptional network promotes anthocyanin biosynthesis in tomato flesh. Mol. Plant.

[56] Zhu Q.L., Yu S., Zeng D., Liu H., Wang H., Yang Z., Xie X., Shen R., Tan J., Li H. (2017). Development of “Purple Endosperm Rice” by engineering anthocyanin biosynthesis in the endosperm with a high-efficiency transgene stacking system. Mol. Plant.

[57] Liu X.Q., Li S.Z., Yang W.Z., Mu B.N., Jiao Y., Zhou X.J., Zhang C.Y., Fan Y.L., Chen R.M. (2018). Synthesis of Seed-Specific Bidirectional Promoters for Metabolic Engineering of Anthocyanin-Rich Maize. Plant Cell Physiol..

[58] Tu M.X., Fang J.H., Zhao R.K., Liu X.Y., Yin W.C., Wang Y., Wang X.H., Wang X.P., Fang Y.L. (2022). CRISPR/Cas9-mediated mutagenesis of VvbZIP36 promotes anthocyanin accumulation in grapevine (*Vitis vinifera*). Hortic. Res..

[59] Koike S., Matsukura C., Takayama M., Asamizu E., Ezura H. (2013). Suppression of g-aminobutyric acid (GABA) transaminases induces prominent GABA accumulation, dwarfism and infertility in the tomato (*Solanum lycopersicum* L.). Plant Cell Physiol..

[60] Li R., Li R., Li X., Fu D., Zhu B., Tian H., Luo Y., Zhu H. (2018). Multiplexed CRISPR/Cas9-mediated metabolic engineering of g-aminobutyric acid levels in *Solanum lycopersicum*. Plant Biotechnol. J..

[61] Kim J.Y., Kim D.H., Kim M.-S., Jung Y.J., Kang K.K. (2024). Physicochemical Properties and Antioxidant Activity of CRISPR/Cas9-Edited Tomato SGR1 Knockout (KO) Line. Int. J. Mol. Sci..

[62] Akama K., Akter N., Endo H., Kanesaki M., Endo M., Toki S. (2020). An In Vivo Targeted Deletion of the Calmodulin-Binding Domain from Rice Glutamate Decarboxylase 3 (OsGAD3) Increases γ-Aminobutyric Acid Content in Grains. Rice.

[63] Nonaka S., Arai C., Takayama M., Matsukura C., Ezura H. (2017). Efficient increase of ɣ-aminobutyric acid (GABA) content in tomato fruits by targeted mutagenesis. Sci. Rep..

[64] Chen C., Zhang M., Zhang M., Yang M., Dai S., Meng Q., Lv W., Zhuang K. (2023). ETHYLENE-INSENSITIVE 3-LIKE 2 regulates β-carotene and ascorbic acid accumulation in tomatoes during ripening. Plant Physiol..

[65] Tripathi L., Ntui V.O., Tripathi J.N. (2024). Application of CRISPR/Cas-based gene-editing for developing better banana. Front. Bioeng. Biotechnol..

[66] Do P.T., Nguyen C.X., Bui H.T., Tran L.T.N., Stacey G., Gillman J.D., Zhang Z.J., Stacey M.G. (2019). Demonstration of highly efficient dual gRNA CRISPR/Cas9 editing of the homeologous GmFAD2-1A and GmFAD2-1B genes to yield a high oleic, low linoleic and α-linolenic acid phenotype in soybean. BMC Plant Biol..

[67] Jarvis B.A., Romsdahl T.B., McGinn M.G., Nazarenus T.J., Cahoon E.B., Chapman K.D., Sedbrook J.C. (2021). CRISPR/Cas9-Induced fad2 and rod1 Mutations Stacked With fae1 Confer High Oleic Acid Seed Oil in Pennycress (*Thlaspi arvense* L.). Front. Plant Sci..

[68] Ludwig Y., Dueñas C., Arcillas E., Macalalad-Cabral R.J., Kohli A., Reinke R., Slamet-Loedin I.H. (2024). CRISPR-mediated promoter editing of a cis-regulatory element of OsNAS2 increases Zn uptake/translocation and plant yield in rice. Front. Genome Ed..

[69] Che J., Yamaji N., Ma J.F. (2021). Role of a vacuolar iron transporter OsVIT2 in the distribution of iron to rice grains. New Phytol..

[70] Ibrahim S., Saleem B., Rehman N., Zafar S.A., Naeem M.K., Khan M.R. (2021). CRISPR/Cas9 mediated disruption of Inositol Pentakisphosphate 2-Kinase 1 (TaIPK1) reduces phytic acid and improves iron and zinc accumulation in wheat grains. J. Adv. Res..

[71] Horton S., Alderman H., Rivera J.A. (2008). Copenhagen Consensus 2008 Challenge Paper Malnutrition and Hunger. https://copenhagenconsensus.com/sites/default/files/CP_Malnutrition_and_Hunger_-_Horton.pdf.

[72] Bouis H., Birol E., Boy E., Gannon B.M., Haas J.D., Low J., Mehta S., Michaux K., Mudyahoto B., Pfeiffer W. (2020). Food Biofortificatio: Reaping the Benefits of Science to Overcome Hidden Hunger.

[73] Vaiknoras K., Larochelle C. (2020). The impact of iron-biofortified bean adoption on bean productivity, consumption, purchases and sales. World Dev..

[74] McNulty M.J., Gleba Y., Tusé D., Hahn-Löbmann S., Giritch A., Nandi S., McDonald K.A. (2019). Techno-economic analysis of a plant-based platform for manufacturing antimicrobial proteins for food safety. Biotechnol. Prog..

[75] Sinke P., Swartz E., Sanctorum H., Van Der Giesen C., Odegard I. (2023). Ex-ante life cycle assessment of commercial-scale cultivated meat production in 2030. Int. J. Life Cycle Assess..

[76] Hefferon K., Swamy B.P.M., Macovei A., Trijatmiko K.R. (2023). Prospects and challenges associated with GM biofortified crops. Genetic Engineering and Genome Editing for Zinc Biofortification of Rice.

[77] De Steur H., Blancquaert D., Strobbe S., Lambert W., Gellynck X., Van Der Straeten D. (2015). Status and market po-ten-tial of transgenic biofortified crops. Nat. Biotechnol..

[78] Mmbando G.S. (2024). The Adoption of Genetically Modified Crops in Africa: The Public’s Current Perception, the Regu-la-tory Obstacles, and Ethical Challenges. GM Crops Food.

[79] Talsma E.F., Melse-Boonstra A., Brouwer I.D. (2017). Acceptance and adoption of biofortified crops in low-and mid-dle-income countries: A systematic review. Nutr. Rev..

[80] Muzhingi T., Langyintuo A.S., Malaba L.C., Banziger M. (2007). Consumer acceptability of yellow maize products in Zimbabwe. Food Policy.

[81] De Groote H., Kimenju S.C. (2012). Consumer preferences for maize products in urban Kenya. Food Nutr. Bull..

[82] Vaiknoras K., Larochelle C., Birol E., Asare-Marfo D., Herrington C. (2019). Promoting rapid and sustained adoption of biofortified crops: What we learned from iron-biofortified bean delivery approaches in Rwanda. Food Policy.

[83] Birol E., Bouis H.E. (2023). Role of socio-economic research in developing, delivering and scaling new crop varieties: The case of staple crop biofortification. Front. Plant Sci..

[84] Katuuramu D.N., Wiesinger J.A., Luyima G.B., Nkalubo S.T., Glahn R.P., Cichy K.A. (2021). Investigation of genotype by environment interactions for seed zinc and iron concentration and iron bioavailability in common bean. Front. Plant Sci..

[85] Avnee, Sood S., Chaudhary D.R., Jhorar P., Rana R.S. (2023). Biofortification: An approach to eradicate micronutrient deficiency. Front. Nutr..

[86] Van Der Straeten D., Bhullar N.K., De Steur H., Gruissem W., MacKenzie D., Pfeiffer W., Qaim M., Slamet-Loedin I., Strobbe S., Tohme J. (2020). Multiplying the efficiency and impact of biofortification through metabolic engineering. Nat. Commun..

[87] Pandey K.B., Rizvi S.I. (2008). Plant Polyphenols as Dietary Antioxidants in Human Health and Disease. Oxidative Med. Cell. Longev..

[88] Malik K.A., Maqbool A. (2020). Transgenic Crops for Biofortification. Front. Sustain. Food Syst..

[89] Cakmak I. (2008). Enrichment of cereal grains with zinc: Agronomic or genetic biofortification?. Plant Soil..

[90] Ishii T., Araki M. (2017). A future scenario of the global regulatory landscape regarding genome-edited crops. GM Crops Food.

[91] Shohael A.M., Hefferon K.L. (2023). Agricultural biotechnology in Bangladesh: The way forward. Agricultural Bioeconomy.

[92] Ahmed S., Shohael A.M., Ahamed T., Ahmed R., Ahmed S., Hassan H.M.S. (2024). Understanding public perspectives on genetically engineered Brinjal and the adoption of modern biotechnology in Bangladesh. Front. Bioeng. Biotechnol..

